# O005. Efficacy of oral supplement compared with amitriptyline in the prophylaxis of episodic tension-type headache and migraine without aura

**DOI:** 10.1186/1129-2377-16-S1-A133

**Published:** 2015-09-28

**Authors:** Biagio Ciccone, Luigi Balzano, Giacinta D'Otolo

**Affiliations:** Ambulatorio ATHENA, Saviano, NA Italy; ASL NA3 SUD, Naples, Italy; Ambulatorio Athena, Saviano, NA Italy

## Introduction

We conducted an observational study of patients attending our outpatient headache clinic, suffering from episodic tension-type headache (ETTH) and migraine without aura (MO). The purpose of the study was to compare the efficacy of magnesium bisglycinate, L-tryptophan, niacin, vitamin B2 and vitamin D, pineal tens (PT) and amitriptyline (A) in the prophylaxis [1–4] of these primary headaches using as outcomes: pain modification with visual analogue scale (VAS); the change in the number of attacks/month; the change in the consumption of analgesics/month.

## Patients and methods

ETTH and MO were diagnosed according to the International Classification ICHD-II criteria. We studied a total of 200 patients: 100 patients were diagnosed with ETTH and 100 with MO. Of these patients, 50 with a diagnosis of ETTH (15 M, 35 F; mean age: 34 years) were treated with PT (1 sachet morning and evening) and were compared with 50 patients (17 M, 33 F; mean age: 39 years) undergoing amitriptyline therapy (20 mg in the evening). Fifty patients with MO (15 M, 35 F; mean age: 37 years) were treated with PT (1 sachet morning and evening), and compared with 50 patients (8 M, 42 F; mean age: 40 years) taking A (20 mg in the evening).

## Results

**Figure 1:**
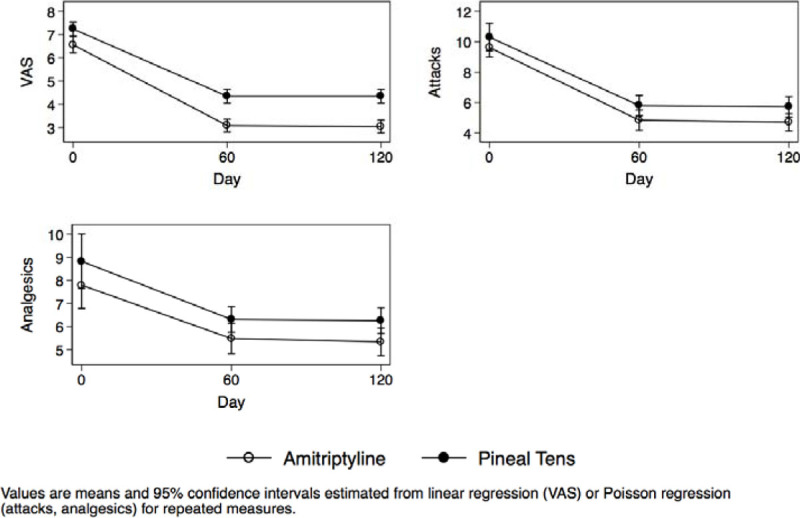
Patients with ETTH.

The VAS modifications, the number of attacks and the number of analgesics taken during the study are shown in Figure 1 for the patients diagnosed with ETTH. The group treated with PT clearly showed a reduction in all treatment outcomes during the study compared to the group taking A.

**Figure 2:**
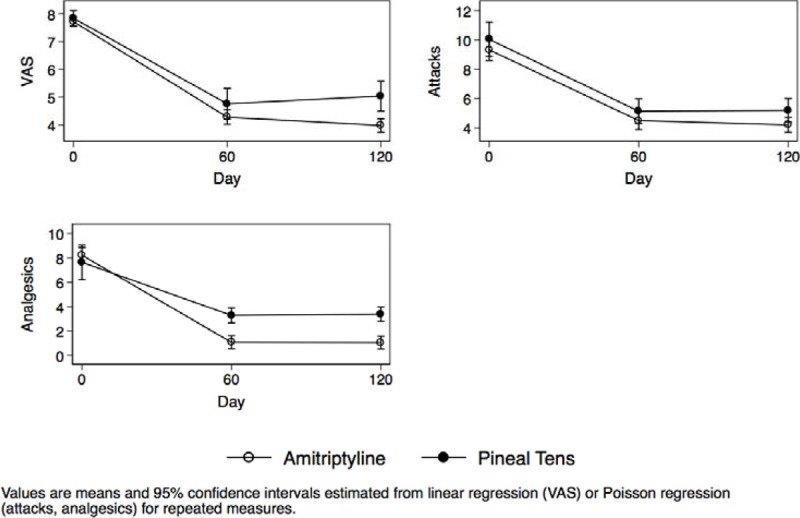
Patients with MO.

VAS modification, the number of attacks and the number of analgesics taken during the study are shown in Figure 2 for the patients diagnosed with MO. The group treated with PT clearly showed a reduction in all treatment outcomes during the study compared to the group taking A.

## Conclusions

Our clinical observation of an improvement in headache in patients receiving PT led us to conduct this cohort study comparing PT with A therapy. Although this study is obviously limited because of the absence of patient randomization, its results confirm the clinical impression of an improvement in the primary headache in patients with PT in terms of improvement in VAS, reduction in the number of attacks/month, and the consumption of analgesics/month. In fact, PT treatment was found to be more efficacious when compared to A treatment in many outcome measures.

Written informed consent to publish was obtained from the patient(s).
